# Microfluidic Droplet Extraction by Hydrophilic Membrane

**DOI:** 10.3390/mi8110331

**Published:** 2017-11-16

**Authors:** Shilun Feng, Micheal N. Nguyen, David W. Inglis

**Affiliations:** 1 School of Engineering, Macquarie University, Sydney, NSW 2109, Australia; michael.nguyen756@gmail.com (M.N.N.); david.inglis@mq.edu.au (D.W.I.); 2 ARC Centre of Excellence for Nanoscale BioPhotonics (CNBP), Macquarie University, Sydney, NSW 2109, Australia

**Keywords:** microfluidic, droplet, extraction, surface tension, membrane

## Abstract

Droplet-based microfluidics are capable of transporting very small amounts of fluid over long distances. This characteristic may be applied to conventional fluid delivery using needles if droplets can be reliably expelled from a microfluidic channel. In this paper, we demonstrate a system for the extraction of water droplets from an oil-phase in a polymer microfluidic device. A hydrophilic membrane with a strong preference for water over oil is integrated into a droplet microfluidic system and observed to allow the passage of the transported aqueous phase droplets while blocking the continuous phase. The oil breakthrough pressure of the membrane was observed to be 250 ± 20 kPa, a much greater pressure than anywhere within the microfluidic channel, thereby eliminating the possibility that oil will leak from the microchannel, a critical parameter if droplet transport is to be used in needle-based drug delivery.

## 1. Introduction

Microfluidic droplets have become a significant field within microfluidics. They have been used for a wide variety of applications where the very small, well-controlled environment of the droplets has enabled new science, new phenomena, and superb repeatability [1]. The ability of droplet microfluidics to transport tiny and precise amounts of fluid over long distances makes it an intriguing improvement over conventional sub-cutaneous drug delivery using micro-needles. 

A conventional micro-needle is useful because it can transport a small amount of fluid into a difficult to reach place. The brain is particularly interesting because neurotransmitter disorders can be highly localised [2]. Conventional microneedles can deliver small amounts of fluid, but have at least two disadvantages that a droplet-based approach could overcome. Firstly, the liquid containing the drug must occupy a large amount of the needle lumen, wasting expensive chemicals. Secondly, it is not possible to change the drug without changing the needle. A droplet based approach to drug delivery could transport a single droplet of drug from a reservoir to the tip without wastage, and a droplet-based approach could entrain two or more droplets containing different drugs for delivery with precise timing. This novel approach has the potential to give unprecedented temporal, spatial, and chemical control to the field of drug delivery. This paper deals with a fundamental obstacle: how to ensure that only the aqueous phase exits the lumen, while the continuous oil phase does not.

Ismagilov [3] demonstrated a microfluidic device with the desired chemical, temporal, and spatial resolution. In this work, micron-sized droplets briefly contacted a confluent cell layer. The droplets mixed with the local environment of the cells in a particular spot, and absorbed local chemical signals and waste products before travelling on. The droplets were transported to state-of-the-art chemical analysis tools giving researchers a clear chemical picture of single cells at regular time points. This chemistrode, as it is called, relies on a fluid seal between the microfluidic channel and the sample, ensuring that there is no net mass transfer between the microchannel and the tissue culture.

Liquid–liquid extraction is of great importance in the preprocessing in microfluidic devices [4].We are interested in a robust method of expelling only the aqueous phase, and this fluid seal prevents the chemistrode from being used to expel droplets. Zeng et al. [5] constructed a device containing a junction between a hydrophobic top channel, carrying water-in-oil droplets, and a narrower hydrophilic bottom channel. When a droplet passed over the bottom channel, an electric field in the bottom channel was able to break the water-oil-water interface, allowing the droplet to coalesce with the fluid in the bottom channel. The application of an electric field to an aqueous phase that is external to the droplet channel is problematic for in vivo use, but may be helpful for use with oils containing surfactants. Others [6] have used electric fields to push droplets across a stream of oil and into a co-flowing aqueous stream. This has been used to extract droplets from a continuous oil phase for mass spectrometry [7]. This approach is not possible where the external aqueous phase is the body of an organism, where the flow and pressure are not controlled and are time-variant.

Wang et al. [8] demonstrated a glass device containing an ‘extraction bridge’ which allowed aqueous droplets to be removed from an oil channel and re-combined for capillary electrophoresis. The extraction bridge consisted of a 6 μm deep hydrophilic channel next to a 70 μm deep, silane-coated channel. The extraction bridge connected one microfluidic system to another and the breakthrough pressure was not measured, but it is not expected to be high due to the comparatively large (6 μm) channel.

Kaigala et al. [9] have achieved precise delivery of fluid using two microfluidic channels, both opening near the same surface. Precise fluid extraction and delivery allows the formation of a small puddle within an immersion liquid. This has been used for liquid patterning. If used for delivery of liquids to an organism, it would extract an unacceptable amount of fluid from the organism. It is also not capable of sequenced delivery of different chemicals. Very recently, Kaigala et al. [10] showed an improved version of the vertical probe that is capable of delivering isolated chemical sequences using immiscible fluid segmentation, what could be referred to as droplets. Their device is also based on restricting fluid flow to parts of the chip by Laplace or capillary pressure. In its two-channel configuration, it bears much functional similarity to our approach, but due to the large pore size is unlikely to have a sufficiently high breakthrough pressure. 

Feng et al. [11] have developed a silicon/glass device capable of water-in oil droplet delivery. It is expensive to fabricate, brittle, and is unlikely to be as suitable medical device. An oleophilic membrane was used to remove the oil phase from water in oil droplets for matrix-assisted laser desorption/ionization (MALDI)-mass spectroscopy by Pereira et al. [12]. 

In this work we show that by placing a hydrophilic membrane over the channel opening, these problems can be overcome. The hydrophilic membrane prevents the oil phase from leaking out of the channel, but allows aqueous droplets to be expelled into an aqueous environment having an unregulated pressure. Placing the membrane port at the end of a needle would allow its use as a precise needle, capable of carrying different chemicals to the same location.

## 2. Theory

At the membrane, oil must be prevented from exiting the device, while acqueous fluidics should pass easily. This is achieved through Laplace pressure, which is given by:

(1) $$\Delta P=\frac{2\tau }{r}\cos(\theta )$$

where τ is the surface tension for the particular fluid interface, r is the radius of the cylinder, and *θ* is the contact angle for the two fluids at the solid boundary. The Laplace pressure required to push air through a wet membrane is called the bubble pressure. The pressure difference between the inside and outside of droplet was descripted in Ding [13].

This concept of a threshold pressure is used here to allow aqueous solutions to exit the channel, while containing the oil phase. Our system uses water and a perfluoropolyether (PFPE) oil, giving two unknown quantities: the surface tension and contact angle. The contact angle is difficult to measure because the membrane is porous, but we are able to experimentally measure the oil-water breakthrough pressure (see the Section 4). 

## 3. Methods

### 3.1. Device Layout and Design

Teh et al. [14] describe various techniques to form microfluidic droplets. These include manipulating the channel geometry and integrating electrodes into microfluidic devices to provide electrical control over droplet generation. Two common methods that generate droplets by manipulation of the channel geometry are T-junctions and flow-focusing. T-junctions can be formed with very large features, and can even be made using a single-step milling process [15].

**Figure 1:**
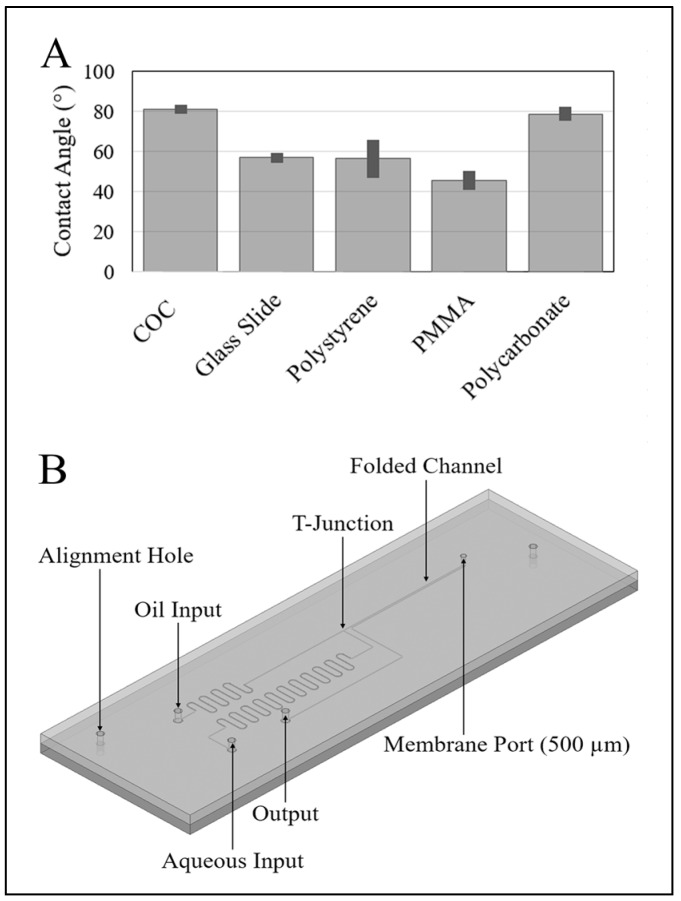
(**A**) Comparison of the water contact angle for potential device materials. Cyclic olefin copolymer (COC) and polycarbonate have the highest contact angle. COC is chosen for soft embossing performance. (**B**) Schematic of droplet delivery device. Note the oil and aqueous inputs, T-junction, and the membrane port.

Water-in-oil droplets are best formed in a hydrophobic channel. We are interested in making the device with a minimum of processing steps so we first investigated common plastics to determine the degree of hydrophobicity. To test this, sub-millimeter water droplets were sprayed onto each surface, and the contact angles of those droplets were measured using an upright microscope where the substrate was held vertically. Multiple droplets were measured for each substrate and averaged to give the results shown in Figure 1A. Error bars show the standard deviation of the measurements. The water droplets on cyclic olefin copolymer (COC) produced the largest contact and so this plastic was chosen. This COC was supplied by microfluidic ChipShop, Jena, Germany (catalog #10-0675-0000-02).

For manufacturing simplicity, our entire channel, including the T-junction, was fabricated using a 100-μm bit on a micromachining mill to a depth of 100 μm (Figure 1B). Upstream of the T-junction are two channels carrying oil and water respectively. Ports were arranged in a 9-mm by 9-mm square, the spacing of a 96 sample well microtiter plate. In order to facilitate a smaller pressure difference at the T-junction, the length of the water channel was made approximately 100 mm long, while the oil channel which carries more viscous fluid was made around 50 mm long.

### 3.2. Hot Embossing of the Microfluidic Channel

The device consists of two COC slides: the bottom slide containing the microfluidic channel, and the top slide containing four ports: oil-input, water-input, output, and membrane. The microfluidic channel was designed in CAD and micro-milled into a 75-mm × 25-mm × 1.5-mm poly methyl methacrylate (PMMA) slide. The channel was then transferred to a COC piece using PDMS-based hot-embossing using a method derived from [16]. Here, a negative poly(dimethylsiloxane) (PDMS) mould was made from the PMMA master channel. The PDMS mould, a blank COC slide, and two glass slide spacers were preheated in a laboratory oven at 158 °C for 10 min. The mould was aligned with the COC, pressed between the two glass slides, and clamped using binder clips. The assembly was placed in a vacuum oven set to 175 °C for 20 min (−98.5 kPa). The assembly was de-moulded while still warm.

### 3.3. Membrane and Lid Bonding

The lid was created from another piece of COC, into which 1-mm input and output port holes were drilled. Alignments holes and a membrane port were also drilled. A surfactant-free cellulose acetate (SFCA) (Sartorious Stedium Biotech AG, Göttingen, Germany) membrane (0.2 μm pore size and 128 μm thick) was laser cut into 1-mm circles. These discs were bonded to the lid, directly over the membrane port using a bonding machine (T-3000-FC3TM, Tresky, Thalwil, Switzerland). A force of 52 g was applied to the membrane, which was rested on a hot plate set at 158 °C for approximately 60 s. Bonding the lid and base was performed as follows: the COC base and lid were assembled using alignment pins, then preheated at 158 °C for 6 min. Using a method that is similar to the embossing method, the lid and the base were placed into the vacuum oven at 160 °C for 10 min. The assembly was annealed at atmospheric pressure and 90 °C for 10 min then left to cool to room temperature.

### 3.4. Droplet Formation

**Figure 2:**
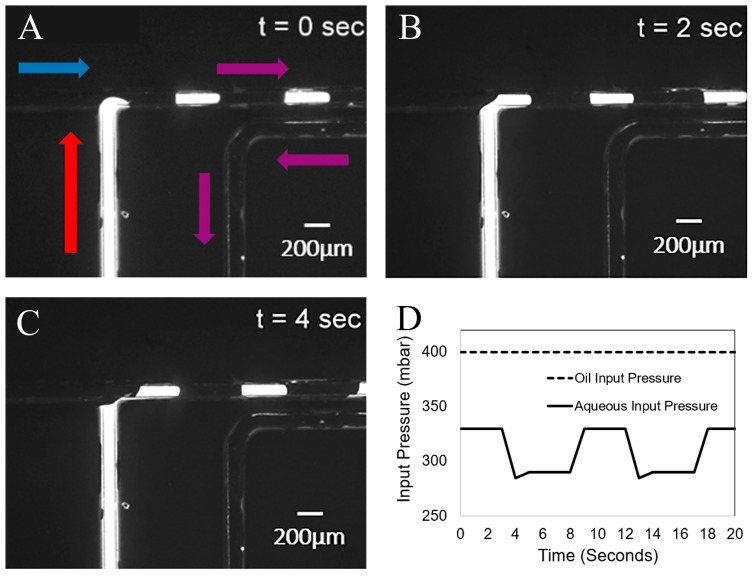
Time step of the creation of rhodamine-solution (red arrow) droplets in oil (blue arrow) at a T-Junction of (**A**) 0 s, (**B**) 2 s, and (**C**) 4 s. Flow direction is indicated by the arrows. (**D**) Plot of input pressures versus time, output, and membrane ports at to atmosphere.

The oil used for the continuous phase was Fomblin 25/6 oil (PFPE) (viscosity of 5.22 Pa·s) and the aqueous phase contained distilled water mixed with diluted rhodamine (viscosity of 1.01 mPa·s). The oil was first passed through the oil input port, filling all channels. The rhodamine water was driven through the water input port until it reached the T-junction. To form droplets on demand, the oil phase pressure was kept constant at 40 kPa, while the aqueous phase pressure was modulated (Figure 2) between 27.5 kPa and 34 kPa. Pressure control was achieved using a Maesflow 4C (Fluigent, Villejuif, France). Operation of the device was observed using an inverted epifluorescent microscope. Figure 2 shows typical droplet formation and the Maesflow pressures used. The average droplet size was 4.5 ± 2.1 nL. At these pressures, it takes approximately 5 s to form a single 5 nL droplet, and a further 30 to 60 s for a droplet to reach the membrane. After a similar time we observe reasonably stable droplet production. Droplets produced at a rate of 0.1 Hz were observed to have an average volume of 4.5 ± 2.1 nL (18 droplets).

## 4. Results

We first established that the membrane, while not being oleophobic, does have a clear preference for water over oil. To do this, coloured water was presented to an oil soaked membrane. A 5-mm diameter piece of membrane was observed to take up the coloured water in a few minutes, becoming entirely coloured and completely expelling the oil. A water-soaked membrane would not absorb oil.

A more quantitative test was then performed to determine the oil breakthrough pressure. We define this as the pressure required to force oil through a wet membrane, overcoming the Laplace pressure of the oil-water interface within the membrane pores. Here, a syringe filter was first wet with coloured water, and then oil was layered onto the high-pressure side. Pressure was slowly increased until flow was observed at the output. We observed that 250 ± 20 kPa (±1 standard deviation) was required to initiate flow (*N* = 3). These pressures are significantly larger than the values used to form droplets. We have been unable to find reported values for the interfacial tension for PFPE oils and water, but liquid hydrocarbon oils and water have tensions of 20–40 dynes per cm (20–40 mN/m). An interface tension of 25 mN/m gives a contact angle of 90°, and no barrier pressure. Interface tensions of 30 and 40 mN/m give water in oil contact angles of 56° and 39°. We can conclude that the interface tension is greater than 25 mN/m, and likely greater than 39°.

**Figure 3:**
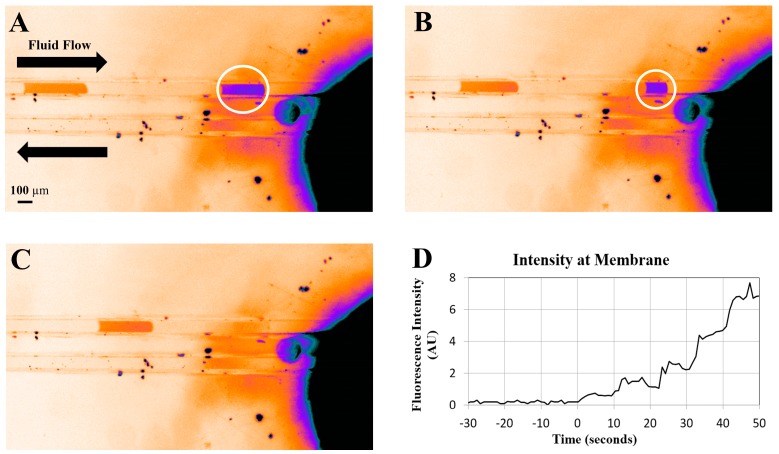
(**A**–**C**) False colour images of droplets flowing into the membrane. The membrane appears black (high intensity) due to staining by rhodamine. Imperfect bonding near the membrane allows the droplet to leak out of the channel before contacting the membrane. (**D**) Total fluorescent intensity over the membrane versus time as rhodamine droplets are delivered. Approximately 5 nL droplets were formed at 10 s intervals. The intensity rises in nearly quantized steps every 10 s, clearly showing regular accumulation of dye at the membrane port while oil continued to flow to the output.

The formation of droplets, separated by oil, is shown in Figure 2. The main achievement in this work is demonstrating that water droplets exit the channel through the hydrophilic membrane, while oil continues to flow out through the microchannel. In order to show that the water droplets only exit through the membrane port and do not flow further towards the oil output port, we observed the accumulation of dyed liquid at the membrane, and no oil, while oil was always observed to flow out of the microfluidic device. Figure 3 shows three images of a fluorescent droplet flowing towards and disappearing in the vicinity of the membrane. Figure 3D shows the fluorescent intensity of the membrane versus time as a series of five droplets arrives at the membrane. Prior to the experiment the membrane is washed externally with water to ensure that it is saturated with water, not oil, and that it has a minimum background level of fluorescence. Droplets were generated every 10 s, and the time axis is shifted such that *t* = 0 coincides with the arrival of the first droplet. We observe that the fluorescence intensity rises in nearly quantized steps every 10 s.

## 5. Discussion

Figure 3 shows an artefact of the bonding process. It is clear that the droplet begins to shrink before it actually reaches the membrane. We believe that this is caused by an un-bonded region surrounding the membrane. The membrane has a thickness of 128 μm, and no cut-out was made for it in the lid. During the bonding process the membrane disk is pressed into the lid with enough pressure and heat to form a seal; however, it still protrudes. This protruding membrane then prevents the lid from contacting and bonding to the bottom substrate in a region of a few hundred microns around the membrane disk. When a droplet that is confined to the channel reaches this area it begins to leak into the space around the membrane; however, this does not appear to significantly affect our ability to measure dye arrival at the membrane.

Throughput and input pressures were low due to the low-cost bonding method used and the high viscosity oil. Thinner oils, such as FC-40, did not produce stable droplets. The use of a higher pressure bonding method will likely result in a more robust device capable of high flow rates.

Droplet formation with this device requires carefully balancing the flows at the T-junction. We find, experimentally, that the pressures required at the inputs for droplet formation change depending on the number of droplets in the channel (between T-junction and membrane). Each droplet adds a significant flow resistance and, hence, a channel with two or three droplets conducts those droplets slower than a channel with just one droplet. The majority of droplet microfluidics use syringe pumps and avoids these problems, but are then unable to produce droplets on demand with a single-layer device.

Linear circuit analysis explains why the pressures needed to generate a droplet may change with the number of droplets in the channel. Using a simple circuit analogue of the microfluidic channels one can calculate the ratio of oil and water flux to the T-junction as a function of input pressures and channel resistances. Assuming a time averaged flow rate that ignores the droplets themselves, this analysis shows that when the input pressures are unequal (as they are in our system), the flux ratio changes non-linearly with downstream resistance. With increasing resistance, one of the fluxes to the junction may reduce to zero, and even reverse direction. 

A stable system can be achieved by having a nearly constant number of droplets moving between the junction and membrane at all times. Repeatedly sending just one droplet, followed by a long delay, is certainly possible using three pressure settings that correspond to: (1) droplet generation; (2) transport to the membrane; and (3) delay. A better approach would be to use local flow control for droplets on demand using any number of techniques, including integrated PDMS valves [17,18] and surface acoustics [19]. These approaches have shown excellent droplet volume and timing control and none of the artefacts we see.

Finally, the observed breakthrough pressure of 250 kPa enables this approach to be used where related work [10] would not. If the membrane port were placed at the end of a microfabricated needle, oil could be driven through the microchannel, carrying droplets at significant speeds and pressures without exceeding the breakthrough pressure.

## 6. Conclusions

In this work we have demonstrated the extraction of aqueous droplets from an oil phase where the aqueous output pressure does not need to be controlled and, in principle, can vary widely. Water-in-oil droplets were observed to exit the microfluidic channel at an integrated hydrophillic membrane while the oil phase continued to flow in the channel. The separation is enabled by a readily available membrane containing 0.2 μm pores that is preferentially wet by water over the continuous oil phase. The wet membrane was observed to be capable of blocking oil penetration up to 250 kPa. In developing a next generation microneedle that harnesses the benefits of droplet based fluid delivery, we must be certain that the continuous phase does not exit the channel. Having a high breakthrough pressure, as we have shown here, is a critical feature, particularly for in vivo applications where oil leakage into tissue is unacceptable and the pressure in the tissue is variable. We believe this work is the first of its kind, and will form an integral part of a new type of microneedle.

## References

[1] Anna S.L. (2016). Droplets and bubbles in microfluidic devices. Annu. Rev. Fluid Mech..

[2] Lee H.J., Son Y., Kim D., Kim Y.K., Choi N., Yoon E.S., Cho I.J. (2015). A new thin silicon microneedle with an embedded microchannel for deep brain drug infusion. Sens. Actuators B.

[3] Chen D., Du W., Liu Y., Liu W., Kuznetsov A., Mendez F.E., Philipson L.H., Ismagilov R.F. (2008). The chemistrode: A droplet-based microfluidic device for stimulation and recording with high temporal, spatial, and chemical resolution. Proc. Natl. Acad. Sci. USA.

[4] Xiong P., Chen X., Xiong Y., Liu G., Tian Y. (2016). Microstructure-enhanced liquid–liquid extraction in a real-time fluorescence detection microfluidic chip. Micromachines.

[5] Zeng S., Pan X., Zhang Q., Lin B., Qin J. (2011). Electrical control of individual droplet breaking and droplet contents extraction. Anal. Chem..

[6] Fidalgo L.M., Whyte G., Bratton D., Kaminski C.F., Abell C., Huck W.T. (2008). From microdroplets to microfluidics: Selective emulsion separation in microfluidic devices. Angew. Chem. Int. Ed..

[7] Fidalgo L.M., Whyte G., Ruotolo B.T., Benesch J.L., Stengel F., Abell C., Robinson C.V., Huck W.T. (2009). Coupling microdroplet microreactors with mass spectrometry: Reading the contents of single droplets online. Angew. Chem. Int. Ed..

[8] Wang M., Roman G.T., Perry M.L., Kennedy R.T. (2009). Microfluidic chip for high efficiency electrophoretic analysis of segmented flow from a microdialysis probe and in vivo chemical monitoring. Anal. Chem..

[9] Kaigala G.V., Lovchik R.D., Drechsler U., Delamarche E. (2011). A Vertical Microfluidic Probe. Langmuir.

[10] Van Kooten X.F., Autebert J., Kaigala G.V. (2015). Passive removal of immiscible spacers from segmented flows in a microfluidic probe. Appl. Phys. Lett..

[11] Feng S., Liu G., Jiang L., Zhu Y., Goldys E.M., Inglis D.W. (2017). A microfluidic needle for sampling and delivery of chemical signals by segmented flows. Appl. Phys. Lett..

[12] Pereira F., Niu X. (2013). A nano LC-MALDI mass spectrometry droplet interface for the analysis of complex protein samples. PLoS ONE.

[13] Ding Y., Qiu F., Casadevall i Solvas X., Chiu F.W.Y., Nelson B.J., deMello A. (2016). Microfluidic-based droplet and cell manipulations using artificial bacterial flagella. Micromachines.

[14] Teh S.Y., Lin R., Hung L.H., Lee A.P. (2008). Droplet microfluidics. Lab Chip.

[15] Nisisako T., Torii T., Higuchi T. (2002). Droplet formation in a microchannel network. Lab Chip.

[16] Goral V.N., Hsieh Y.C., Petzold O.N., Faris R.A., Yuen P.K. (2011). Hot embossing of plastic microfluidic devices using poly(dimethylsiloxane) molds. J. Micromech. Microeng..

[17] Galas J.C., Bartolo D., Studer V. (2009). Active connectors for microfluidic drops on demand. New J. Phys..

[18] Choi J.W., Lee S., Lee D.H., Kim J., Chang S.I. (2014). Integrated pneumatic micro-pumps for high-throughput droplet-based microfluidics. RSC Adv..

[19] Collins D.J., Alan T., Helmerson K., Neild A. (2013). Surface acoustic waves for on-demand production of picoliter droplets and particle encapsulation. Lab Chip.

